# In-host adaptation of *Staphylococcus aureus* during recurrent prosthetic joint infections: a retrospective longitudinal study

**DOI:** 10.1128/spectrum.04225-25

**Published:** 2026-09-16

**Authors:** Charles Maurille, François Gravey, Jean-Christophe Giard, Jocelyn Michon, Goulven Rochcongar, Bertrand Lecoeur, Renaud Verdon, Simon Le Hello, Christophe Isnard

**Affiliations:** 1 Department of Infectious Diseases, Normandie Univ, UNICAEN, UNIROUEN, CHU de Caen Normandie, INSERM U1311 DynaMicURe, CAEN, France; 2 Department of Infectious Diseases, Centre Hospitalier du Mans, Le Mans, France; 3 Department of Infectious Diseases, CHU Angers, Angers, France; 4 Department of Infectious Agents, Bacteriology Service, Normandie Univ, UNICAEN, UNIROUEN, CHU de Caen Normandie, INSERM U1311 DynaMicURe, CAEN, France; 5 Normandie Univ, UNICAEN, UNIROUEN, INSERM U1311 DynaMicURe, CAEN, France; 6 Department of Infectious Diseases, Normandie Univ, UNICAEN, CHU de CAEN Normandie, CAEN, France; 7 Department of Orthopaedic and Trauma Surgery, Normandie Univ, UNICAEN, CHU de CAEN Normandie, CAEN, France; Meijo University, Nagoya, Japan

**Keywords:** genomic, virulence, recurrence, prosthetic joint infections, *Staphylococcus aureus*

## Abstract

The aim of this study was to characterize the *in vivo* evolution of *Staphylococcus aureus* strains involved in recurrent prosthetic joint infections (PJIs) both phenotypically and genomically. We conducted a monocentric retrospective study in a 1,437-bed French teaching hospital between 2013 and 2021. All patients presenting a recurrent *S. aureus*-related PJI—defined as at least two strains isolated from distinct clinical samples more than 90 days apart—of the knee, hip, or shoulder were included. Clinical data were reviewed, and all isolates underwent phenotypic characterization, including antimicrobial susceptibility testing, growth rate determination, biofilm production assays, metabolic profiling (API 50 CH), and virulence evaluation using the *Galleria mellonella* infection model. Whole-genome sequencing (WGS) was performed for all strains, followed by analyses of core-genome multilocus sequence typing (cgMLST), resistome, virulome, and mobilome composition, and single-nucleotide polymorphisms (SNPs). Thirteen patients met inclusion criteria, yielding 55 *S. aureus* isolates. Eight patients experienced recurrent infections caused by genetically closely related strains throughout the clinical course (median: three strains per patient; range: 2–6), whereas five patients were infected by genetically distinct strains. At baseline, isolates were genetically diverse and susceptible to methicillin and rifampicin; two showed fluoroquinolone resistance due to *grlA* and/or *gyrA* mutations. In one patient (patient C), a recurrent isolate acquired an *rpoB* S486L mutation, conferring rifampicin resistance after rifampicin exposure. Due to the limited sample size, it is difficult to draw definitive conclusions from the phenotypic analyses. This study highlights the adaptive evolution of *S. aureus* during chronic PJIs and underscores the need for further research to better understand intra-host dynamics in long-standing infections.

**IMPORTANCE:**

This study conducted in a 1,437-bed French teaching hospital analyzed the genomic and phenotypic evolution of 55 *Staphylococcus aureus* strains recovered in recurrent PJIs from 13 patients. The first strains showed high genotypic diversity across 12 different sequence types. Among the 13 patients, only eight experienced a true recurrence with the same strain, while five were contaminated with a different strain of *S. aureus*, indicating a new infection. Moreover, this study underscores the complex within-host evolution of *S. aureus* and highlights the phenotypical and genotypical adaptation during chronic infection.

## INTRODUCTION

Prosthetic joint infection (PJI) is a devastating complication of arthroplasty posing a significant social burden and leading to increased morbidity and mortality (1). The successful management of PJIs requires a coordinated approach involving surgical intervention and antimicrobial therapy (2). Despite extensive knowledge about PJI management, over 20% of patients experience recurrent or persistent infections (3). The mechanisms underlying treatment failure are not well understood but are likely multifactorial. One potential factor could be related to the intrinsic characteristics of the pathogen and its ability to adapt to the host environment (4, 5).

*Staphylococcus aureus* is one of the leading pathogens accounting for over a quarter of PJIs (4). This species is both a skin commensal, with approximately 35% of the human population being nasal carriers (6), and an opportunistic pathogen that can cause a wide range of clinical infections, including endocarditis, pneumonia, skin and soft tissue, or osteoarticular infections (7). *S. aureus* exhibits a diverse genetic background resulting from both vertical and horizontal evolution (8). This latter significantly contributes to the adaptation of *S. aureus* through the transfer of mobile genetic elements, facilitating the rapid acquisition of virulence factors or antimicrobial resistance genes (8). During chronic infections, such as PJIs, bacterial populations have to cope with evolutionary forces, such as host immune response, antimicrobial therapy, competition with other micro-organisms, physical barriers to colonization, or nutritional deficiencies. These selective pressures promote the selection of genetic variants based on their ability to survive and proliferate in a new niche (9).

Compared with experimental studies, observing the natural evolution of intra-host bacterial populations incorporates multiple uncontrolled variables, thereby better reflecting the complexity and diversity of life. Most human studies based on longitudinal genomic analysis of microorganisms have been conducted in cystic fibrosis patients with chronic lower respiratory tract infections (9). However, each tissue represents a unique microenvironment, leading to distinct bacterial evolution and specific host-pathogen interactions. In bone and joint infections, the reduced accessibility of the infected site for sampling has limited the number of studies investigating the phenotypic and molecular adaptive process of *S. aureus* during long-term persistence (10–14). The aim of this study was to describe, at both phenotypic and genomic levels, the within-host evolution of *S. aureus* isolated from recurrent PJIs.

## MATERIALS AND METHODS

### Setting and patients

We conducted a retrospective cohort study at the Caen University Hospital (France), a 1,437-bed teaching hospital in Normandy, France, and one of the reference centers for complex bone and joint infections in the country (15). All patients with recurrent PJIs on the knee, hip, and shoulder caused by *S. aureus* from January 2013 to December 2021 were included. PJI was defined according to the Musculoskeletal Infection Society (MSIS) criteria (16). A recurrent infection was defined by the presence of a bacterial strain that was identical at the species level on at least two different deep samples separated by an interval of more than 90 days, considered as delayed or late infections (2). This interval selected allows sufficient time for genomic and phenotypic adaptation. Two strains were considered genetically closely related when isolated from the same patient and sharing the same ST and *spa* type. We defined a true recurrent-PJI (TR-PJI) when the bacteria isolated during the first and second were genetically closely related and a false recurrent-PJI (FR-PJI) when the bacteria were non-isogenic. Management of PJIs was decided during a multidisciplinary meeting with infectious disease specialists, orthopedic surgeons, and microbiologists following the Philadelphia consensus conference and Infectious Diseases Society of America (IDSA) guidelines (2, 17). The data related to clinical characteristics, time between surgery and failure, surgical management, and antimicrobial therapy were recorded using the informatics medical records (Usv2-Crossway, McKesson, Philadelphia, CA, USA). PJIs were classified according to the Tsukayama classification: early postoperative infection (<4 weeks), late chronic infection (>4weeks), or acute hematogenous origin (18). Raw characteristics were compared between groups using Fisher’s exact test for quantitative variables and Student’s *t* or Mann-Whitney *U* test for quantitative variables. A *P* value less than 0.05 was considered significant and indicated in bold; all *P* values were two-tailed. To estimate the prevalence rate of PJIs, we used the ratio of patients with a documented diagnosis of PJI to the total number of patients who received an arthroplasty during the study period. The study was conducted in accordance with the Declaration of Helsinki and approved by the Ethics Committee of the Biomedical Research Institute of Caen (ID 3084, 02/15/2022). In accordance with French regulations, patients were informed of the use of their medical and biological data for research purposes and did not object to their use in this study.

### Bacterial strains, growth conditions and identification

The standard microbiological protocol includes the culture of different anatomic samples during routine patient care and according to current standards. Briefly, all samples were prospectively inoculated on the following media: blood agar, PVX agar, and Schaedler broth with adjunction of vitamin K3, all from bioMérieux (Marcy l’Etoile, France) under different atmosphere conditions (e.g., anaerobic and aerobic conditions). Biopsies were mechanically ground before their inoculation. Sonication of the removed implants was not used. All cultures performed in agar media were incubated for 5 days, whereas Schaedler broth cultures were incubated for 15 days. Identification was confirmed by matrix-assisted laser desorption/ionization time-of-flight (MALDI-TOF) mass spectrometry (Microflex LH/SH analyzer, Brucker, Bremen, Germany). The initial strains were stored in glycerol-supplemented brain heart infusion broth at room temperature. The samples were then subcultured on blood agar plates. After incubation, colonies were isolated and grouped based on their distinct macroscopic characteristics, such as colony size, color, and hemolytic pattern. This approach enabled differentiation of strains according to their specific phenotypic profiles. After inclusion in our study, strains were stored at −80°C in 15% glycerinated in-house brain heart infusion. For all genomic and phenotypic experiments, bacterial strains were subcultured in a single step from the original frozen stock to ensure colony homogeneity and allow consistent association between genotypic and phenotypic characteristics.

### Whole-genome sequencing and bioinformatics

DNA was extracted from pure bacterial overnight cultures on TS broth using NucleoSpin Microbial DNA (Macherey-Nagel, Düren, Germany) following the manufacturer’s instructions. Before storage at −80°C, RNase A treatment was performed to remove residual RNA. The concentration and quality of the DNA were assessed by Quantus fluorometer (Promega, Madison, WI, USA) and NanoDrop spectrophotometer (Thermo Fisher, Waltham, MA, USA). Then, we conducted whole-genome sequencing of all strains using the NextSeq 500 System (Illumina, San Diego, CA, USA) at the Plateforme de Microbiologie Mutualisée (P2M) of the Institut Pasteur (Paris, France), generating 150 base paired-end reads. The nucleotide sequence data have been deposited in GenBank with the BioProject accession number PRJNA1182127. Alien Trimmer software was used for read trimming and clipping. The quality of the filtered fastq files was evaluated using FastQC software. Genomes were *de novo* assembled using SPAdes v. 3.12 software with the recommended kmer sizes for Illumina 150 bp paired reads (-k 21, 33, 55, and 77) (19). The quality of the genome assembly was assessed using the Quast tool (20). Species confirmation based on 16S rRNA, determination of multilocus sequence typing (MLST), and *spa* typing were performed using the Center for Genomic Epidemiology website (21–23). *In silico* detection of resistance genes or chromosomal point mutations associated with phenotypic resistance, virulence factors, and prophage DNA detection was carried out using ResFinder v 4.1 (24, 25), VirulenceFinder v 2.0 (26), and PHASTER (27). Core genome MLST (cgMLST) was performed using chewBBACA (github.com/B-UMMI/chewBBACA) with the *Staphylococcus aureus* scheme available online (https://www.cgmlst.org/ncs/schema/Saureus/). For the determination of new alleles in our strains, the script has been run twice.

High-resolution genomic distance matrices were generated using the Snippy pipeline (v4.6.0) by mapping assemblies against species-specific closed reference genomes. Strict core-genome single nucleotide polymorphism (cgSNP) cutoffs were applied to define clonal identity (e.g., cgSNP ≤ 20 with 10 min cov, one reference strain per ST, and an average Ref_length of 2,786,786 (28).

### Antimicrobial susceptibility testing

Antimicrobial susceptibility testing (AST) was performed on agar medium according to the guidelines of the “Comité de l’antibiogramme de la société Française de microbiologie” in association with the European Committee on Antimicrobial Susceptibility Testing (CA-SFM/EUCAST) as of April 2021 v 1.0 (https://www.sfm-microbiologie.org/2021/04/23/casfm-avril-2021-v1-0/). The antibiotics tested included penicillin G, cefoxitin, moxifloxacin, tetracyclines, erythromycin, clindamycin, quinupristin/dalfopristin, linezolid, kanamycin, tobramycin, gentamicin, fusidic acid, trimethoprim-sulfamethoxazole, rifampicin, ofloxacin, levofloxacin, and fosfomycin. Minimum inhibitory concentrations (MICs) in microdilution were carried out on a Sensititre plate (Thermo Fisher, Waltham, MA, USA) according to the manufacturer’s instructions. The panel included ceftaroline, ceftobiprole, clindamycin, dalbavancin, daptomycin, levofloxacin, linezolid, oxacillin, rifampicin, tedizolid, teicoplanin, trimethoprim/sulfamethoxazole, and vancomycin. After 24 h of incubation, the plates were read with plate reader (Vizion, Thermo Fisher, Waltham, MA, USA), looking for turbidity as an indicator of microbial growth. MICs values were determined as the lowest concentration of antibiotic inhibiting microbial growth. All results were interpreted according to CA-SFM/EUCAST 2024 breakpoints (https://www.sfm-microbiologie.org/2021/04/23/casfm-avril-2021-v1-0/).

### Growth rate determination

After adjusting the optical density measured at 600 nm (OD_600_) to 1.00 ± 0.05 from an overnight culture in tryptone soy (TS) broth medium, the bacterial suspensions were diluted 1:100 in TS. These bacterial suspensions were used to fill the wells of a 96-well flat-bottomed sterile polystyrene microplate (CytoOne, StarLab, Humburg, Germany). The plate was incubated with shaking every 8.5 min for 15 h at 37°C. OD_595_ was measured every 20 min using the microtiter plate reader Tecan Infinite 200 Pro (Tecan, Männedorf, Switzerland). The intrinsic growth rate, the doubling time, and the empirical area under the curve were estimated with the Growthcurver package implemented in R v 4.2.1 software (29). *P* values were determined using Mann-Whitney U-test.

### Biofilm formation

Biofilm formation study by the crystal violet staining was assessed as previously described by Stefanovic et al. (30). Briefly, after adjusting the optical density measured at 600 nm (OD_600_) to 1.00 ± 0.05 from an overnight culture in tryptone soy (TS) broth medium, the bacterial suspensions were diluted 1:100 in sterile TS with 1% glucose (D-(+)-glucose, Sigma-Aldrich, Saint-Quentin-Fallavier, France). Then, 100 µL of each suspension was incubated in flat-bottomed 96-well polystyrene microtiter plates (Sarstedt, Nümbrecht, Germany). After 24 h ± 30 min of static incubation at 35 ± 2°C, the culture broth was then gently removed, and plates were washed three times with 200 μL of phosphate-buffered saline (PBS) (Sigma-Aldrich). The biofilm formed by the adherent bacteria was fixed using 100 μL of 99% methanol (VWR, Rosny-Sous-Bois, France) per well for 20 min and air-dried for 1 h under a chemical hood. Rinse with sterile water 100 μL twice. The fixed bacteria were stained with 100 μL of 1% crystal violet (Sigma-Aldrich) per well for 10 min. The excess dye was rinsed off by washing five times with 200 μL of water before air-drying under hood for 1 h. The biofilm-bound dye was resolubilized with 100 μL of 33% glacial acetic acid (Fischer Scientific, Illkirch-Graffenstaden, France) per well. OD_595_ was measured with the microtiter plate reader Tecan Infinite F200 Pro (Tecan, Männedorf, Switzerland). Each assay was performed in triplicate in three independent experiments. Negative control wells were prepared with TS with 1% glucose alone. The *S. aureus* strain ATCC 29213 was used as a control. The intensity of crystal violet staining was expressed relative to the OD_595_ measured after 24 h of growth in three independent experiments (biofilm: OD_biofilm_ / OD_growth_). The *P* value was determined with a Mann–Whitney *U* test.

### Metabolism study on the API50CH gallery

The fermentation capacity of carbohydrates and derivatives was assessed on API50CH galleries (bioMérieux, Marcy-l'Etoile, France). All strains were inoculated into API50CHL media (bioMérieux, Marcy-l'Etoile, France) at 2 MacFarland. Bacteria in suspension were transferred to each of the 50 API50CH wells. The strips were incubated at 37°C aerobically for 48 h. Color changes reflecting acidification of the medium by the fermentation of a substrate were evaluated after 24 and 48 h of incubation. The *S. aureus* strain ATCC 29213 was used as a control. Results were plotted using the ggplot package with R v 4.2.1 software. The *P* value was determined with a Wilcoxon signed-rank test.

### *Galleria mellonella* larva virulence model of infection

Overnight bacterial cultures in TS broth were centrifuged, and pellets were washed three times with sterile NaCl 0.9% and resuspended at an OD_600_ of 0.5. For each experiment, 10 larvae weighing around 150 mg and in the last stage were infected subcutaneously with 10 μL of cell suspensions of each strain via their last proleg using microfine 1-mL insulin syringes (Terumo, Leuven, Belgium) with a syringe pump (kdScientific, Holliston, MA, USA). They were incubated at 35 ± 2°C, and larval mortalities were then monitored at 1, 2, and 3 days post-infection. As a control, PBS was injected into 10 larvae for each experiment. If a total of two larvae from PBS groups died, the experimental results were discarded. Experiments were repeated independently three times. Inoculum sizes were measured by serial dilutions and colony-forming unit (CFU) plating from the inoculum of each experiment. Results were represented on a Kaplan-Meier survival curve, and the log-rank test was conducted with R v 4.2.1 software.

## RESULTS

### Genotyping and phylogeny analysis

**Fig 1:**
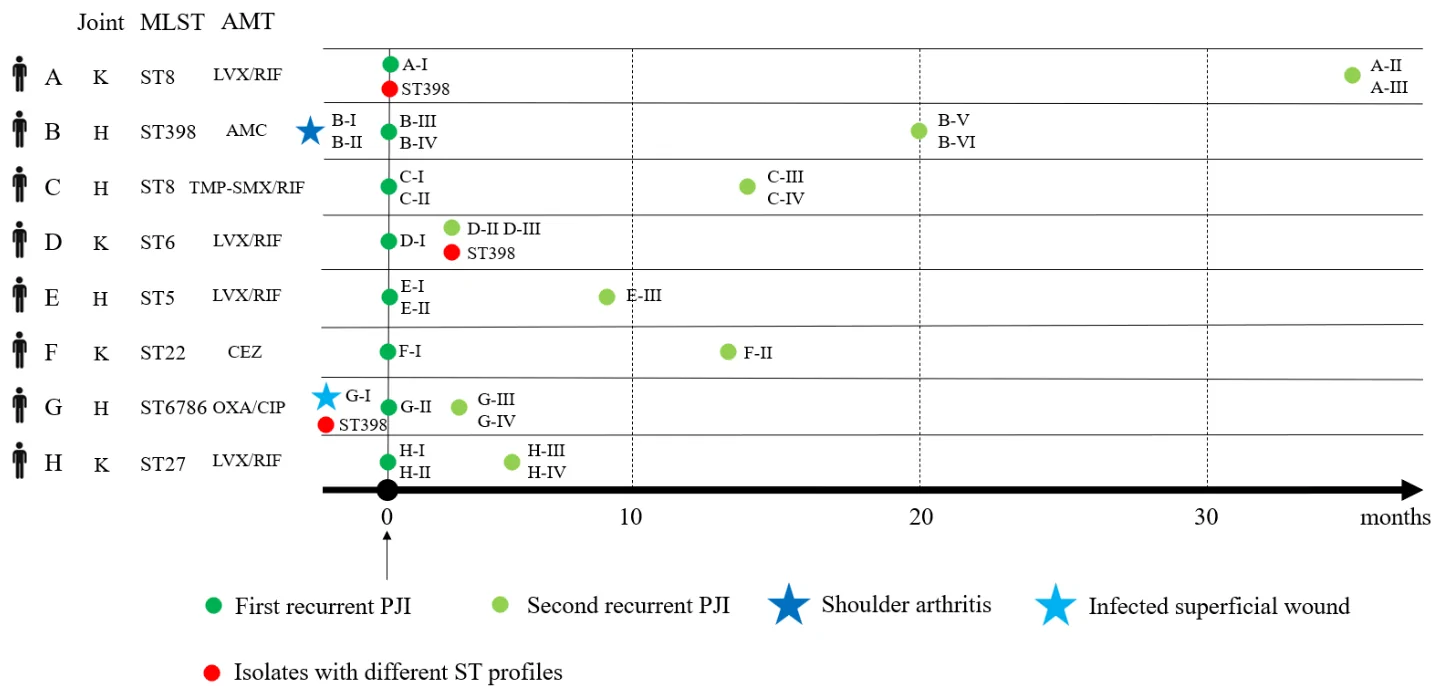
Schematic representation of the clinical course and strains isolated in the eight patients with true recurrent-PJI (abbreviations: AMC, amoxicillin-clavulanic acid; AMT, antimicrobial therapy; CEZ, cefazolin; CIP, ciprofloxacin; DAIR, debridement antibiotics and implant retention; H, hip; K, knee; LVX, levofloxacin; MLST, multilocus sequence typing; OXA, oxacillin; PJI, prosthetic joint infection; RIF, rifampicin; TMP-SMX, trimethoprim-sulfamethoxazole).

**Fig 2:**
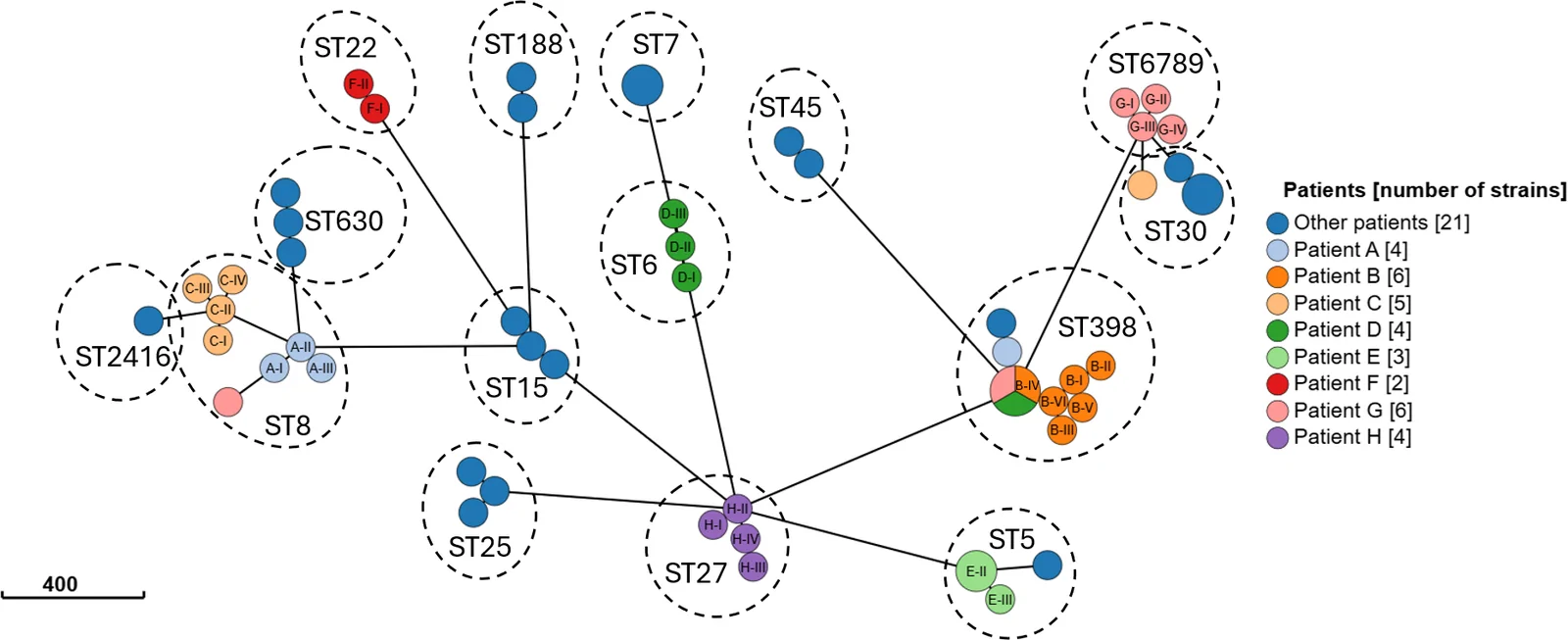
Minimum-spanning tree based on the core genome of 55 isolates included in the study. Phylogeny analysis was generated using the GrapeTree freely available module.

Over the study period, the prevalence of PJI at our institution was 1.98% for knee arthroplasties and 2.14% for hip arthroplasties. Thirteen patients met the inclusion criteria of *S. aureus*-related recurrent PJI by an interval of more than 90 days on the knee, hip, or shoulder. A total of 55 *S. aureus* strains were included. The initial PJI strains belonged to 12 different sequence types (STs) (ST5, ST6, ST8, ST15, ST22, ST25, ST27, ST30, ST45, ST188, ST398, ST6786), indicating high genotypic diversity. Only two patients shared the same sequence type (ST8) of *S. aureus*-related recurrent PJI, and two patients had a close ST type, ST30 and its single-locus variant ST6786. Eight patients had true recurrent PJIs (TR-PJI) with strains belonging to the same ST and *spa* type in each patient, for a total of 29 strains (median per patient: 3; range: 2–6) (Fig. 1). Twelve *S. aureus* strains were from the first TR-PJIs, 14 from the second, and three from other sites. Among these three strains, two were isolated from previous shoulder infection prior to the first TR-PJIs (patient B, strains B-I and B-II), and one strain was isolated from an infected superficial wound before the first TR-PJI (patient G, strain G-I), suggesting hematogenous and contiguous spread, respectively. Additionally, non-clonally related *S. aureus* strains belonging to ST398 were found in patients A, D, and G during the clinical course of TR-PJIs but were not identified before or after these episodes. Phylogenetic analysis of the 55 core genomes (draft assemblies) confirmed the high genotypic diversity between patients (Fig. 2). Chromosomal mutations of fewer than 20 core SNPs (ranging from 2 to 17) were observed among TR-PJI strains collected between 3 and 35 months apart (Table S1). Moreover, the SNPs’ variation among ST398 strains isolated from patients B, D, and G, as well as from a patient with an FR-PJI, was fewer than eight core SNPs (ranging from 0 to 8), suggesting the existence of a cluster among these patients.

### Clinical data of patients with TR-PJIs

Infections were classified as early postoperative infections (<4 weeks) for patients A and E, late chronic infections (>4 weeks) for patients B, C, and F, and acute hematogenous for patients D, G, and H (Table 1). Seven patients experienced relapse despite appropriate surgery and antimicrobial therapy, while one patient had a persistent infection under a chronic oral antimicrobial suppression regimen consisting of amoxicillin-clavulanate due to the inability to undergo surgery (patient B). The median interval between the first and second recurrent PJIs was 10.5 months (ranging from 3 to 35 months). Debridement, antibiotics, and implant retention (DAIR) were performed in five patients, a one-stage exchange in patient F, and a two-stage exchange in patient C. Antimicrobial therapy regimens included fluoroquinolones for five patients, rifampicin for five patients, trimethoprim-sulfamethoxazole for one patient, cefazolin for one patient, and oxacillin for one patient. Compared with TR-PJIs, two-stage exchange was significantly more frequent in FR-PJI cases (*P* = 0.03; Table S2). Antibiotic therapy lasted 12 weeks for three TR-PJI patients (38%) and all five FR-PJI patients (100%), whereas a 6-week course was administered to 5 TR-PJI patients (62.5%) and none of the FR-PJI patients (*P* = 0.08). No other significant clinical differences were identified from the remaining data in either group.

**TABLE 1:** Clinical characteristics, management, and outcome of patients included in the study^*c*^

Patients	A	B	C	D	E	F	G	H
Sex, age (years)	F, 72	F, 83	H, 43	H, 59	F, 77	H, 75	H, 64	F, 83
Comorbidity	Obesity	NA	NA	Chronic heart failure	Ischemic stroke	Chronic heart failure, cirrhosis	Chronic heart failure, cirrhosis	Chronic kidney disease
Affected joint	Knee	Hip	Hip	Knee	Hip	Knee	Hip	Knee
No of surgeries prior to first recurrent PJI	4	1	1	3	1	3	1	1
Type of infection^*a*^	Early	Chronic	Chronic	Hematogenous	Early	Chronic	Hematogenous	Hematogenous
Surgical treatment^*a*^	DAIR	NA	2-stage exchange	DAIR	DAIR	1-stage exchange	DAIR	DAIR
Antibiotics^*a*^	LVX/RIF	AMC	TMP-SMX/RIF	LVX/RIF	LVX/RIF	CEZ	OXA/CIP	LVX/RIF
Duration of antibiotics (days)^*a*^	42	∞^*b*^	73	42	42	84	42	84
Outcome^*a*^	Relapse	Persistence	Relapse	Relapse	Relapse	Relapse	Relapse	Relapse
MLST	ST8	ST398	ST8	ST6	ST5	ST22	ST6786	ST27
*spa* type	t24	t571	t8	t701	t67	t608	t12	t193

^*a*^ Of the first recurrent PJI.

^*b*^ ∞, indefinite chronic antibiotic suppression regimen.

^*c*^ AMC, amoxicillin-clavulanic acid; CEZ, cefazolin; CIP, ciprofloxacin; DAIR, debridement antibiotics and implant retention; LVX, levofloxacin; MLST, multilocus sequence typing; OXA, oxacillin; RIF, rifampicin; TMP-SMX, trimethoprim-sulfamethoxazole; NA, non applicable or unknown.

### Antimicrobial susceptibility testing of strains recovered in TR-PJIs

Among strains isolated at baseline from patients with TR-PJIs, antimicrobial resistance profiles were largely non-resistant to antibiotics tested (Table 2). All strains were susceptible to methicillin and rifampicin. Five strains were penicillin-resistant and harbored a *blaZ* gene coding for staphylococcal ß-lactamase; one was resistant to erythromycin and carried *erm(A)* gene; one was classified as susceptible, increased exposure to erythromycin and harbored *erm(T)* gene; and two were resistant to fluoroquinolones with a point mutation in *grlA* and/or *gyrA* genes. Throughout the clinical course, no changes in antibiotic resistance were detected, except for patients B and C. In patient C, who was treated with rifampicin during the first recurrent TR-PJI, the corresponding strain exhibited a chromosomal point mutation leading to amino acid substitution in position 486 of RpoB protein (S486L), conferring phenotypic resistance to rifampicin (Fig. S1a). For patient B, erythromycin susceptibility decreased between strains B-I/B-II compared with strains isolated after (Fig. S1b). *In silico* analysis using ResFinder 4.6.0 database (http://genepi.food.dtu.dk/resfinder) did not identify the acquisition of additional antimicrobial resistance gene in these strains. Additionally, analysis of *erm(T)*, RNAr23S, L3, L4, and L22-encoding genes, and promoters did not show differences between strains that were susceptible with increased exposure and resistant strains (data not shown).

**TABLE 2:** Phenotypic and genotypic antimicrobial resistance of 12 isogenic strains isolated during the first recurrent PJI^*b*^

Isolates^*a*^	Phenotypic resistance	Phenotypic susceptible, increased exposure	Resistance gene	Chromosomal point mutations
A-I	PNG	NA	*blaZ*	NA
B-III/B-IV	PNG	ERY	*blaZ*, *erm(T*)	NA
C-I/C-II	CMN, ERY, OFX	NA	*ant (9)-Ia*, *erm(A*)	*grlA* (Ser8-Phe)
D-I	WT	NA	NA	NA
E-I/EII	WT	NA	NA	NA
F-I	OFX, PNG	NA	*blaZ*	*grlA* (Ser8-Phe), *gyrA* (Ser84Leu)
G-II	PNG	NA	*blaZ*	NA
H-I/H-II	PNG	NA	*blaZ*	NA

^*a*^ Recovered from the first recurrent PJI episode.

^*b*^ CMN, clindamycin; ERY, erythromycin; OFX, ofloxacin; PNG, penicillin G; WT, wild-type phenotype; NA, non applicable.

### Virulome and mobilome analysis of TR-PJIs

**Fig 3:**
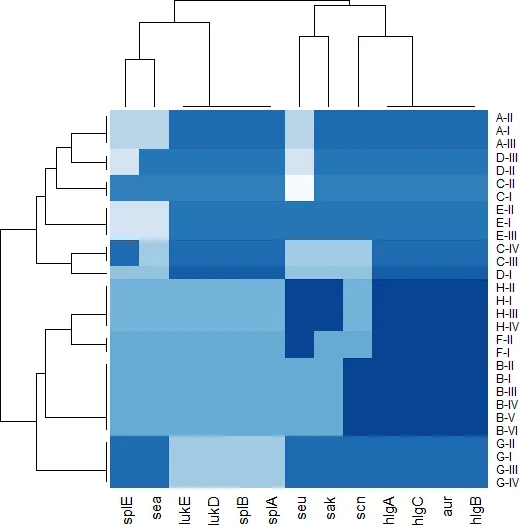
Clustered genotypic analysis of virulome of 55 isolates included in the study. *In silico* analysis of virulome was performed using VirulenceFinder v 2.0. Cluster analysis was generated using R v 4.2.1 software. Darker blue shading indicates the presence of a virulence gene, whereas lighter shades indicate its absence. Each letter (A–H) represents a patient, and each Roman numeral associated with a given letter represents a distinct isolate recovered from that patient. The heatmap illustrates the distribution of virulence-associated genes among the 55 sequential isolates recovered from eight patients. Hierarchical clustering revealed that isolates from the same patient generally exhibited highly similar virulence gene profiles, with only minor variations observed in a limited number of genes, particularly sea, sak, and scn for patients C and D. Overall, the virulome remained highly conserved within each patient over time.

As presented in Fig. 3, all strains clustered according to each patient, except for patients C and D, who exhibited modification in virulence genes. For patient C, the *scn*, *sak*, and *sea* genes were lost between the first and second PJIs, while patient D gained these same genes. These virulence genes constitute an immune evasion cluster (IEC) encoded by a prophage known as SA345ruMSSAST8. The genome sizes of these phages were 50.9 kb in strains C-I and C-II and 30.8 kb in strains D-II and D-III. The genome of SA345ruMSSAST8 isolated in strains C-I, C-II, D-II, and D-III contained 32 and 25 open reading frames (ORF), respectively. The WGS of another strain (ST398) isolated during the second recurrent TR-PJI in patient D revealed only the presence of the *scn* gene harbored on a different prophage (ΦSa3 phage P282), excluding potential horizontal gene transfer. PHASTER analysis also detected the partial acquisition of φNM3 in recurrent strains from patient D, specifically carrying *lukS-PV* and *lukF-PV* genes.

### Phenotypic analysis of TR-PJIs

**Fig 4:**
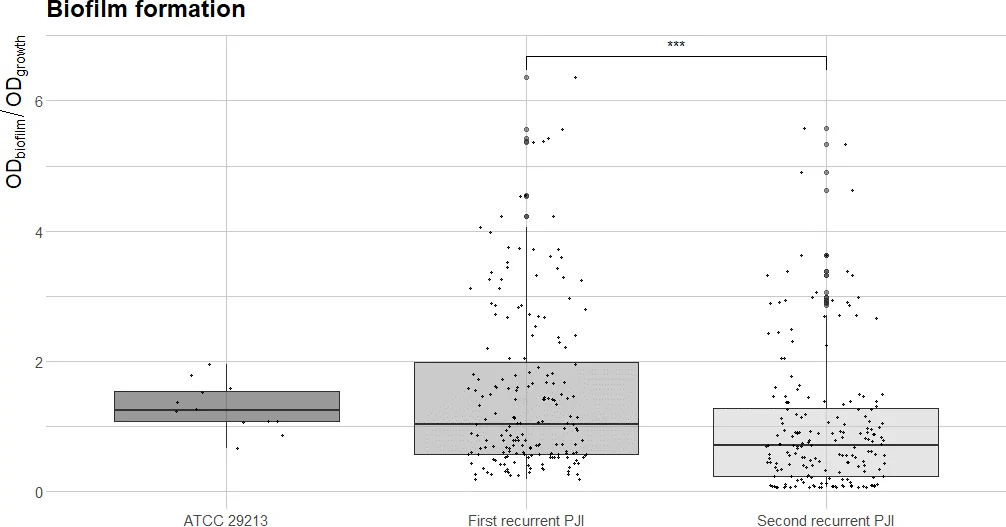
Biofilm formation using crystal violet staining by strains isolated during the first recurrent PJI and during the second recurrent PJI. Each point represents one experiment. Error bars represent the standard deviations of three independent experiments for each strain.

**Fig 5:**
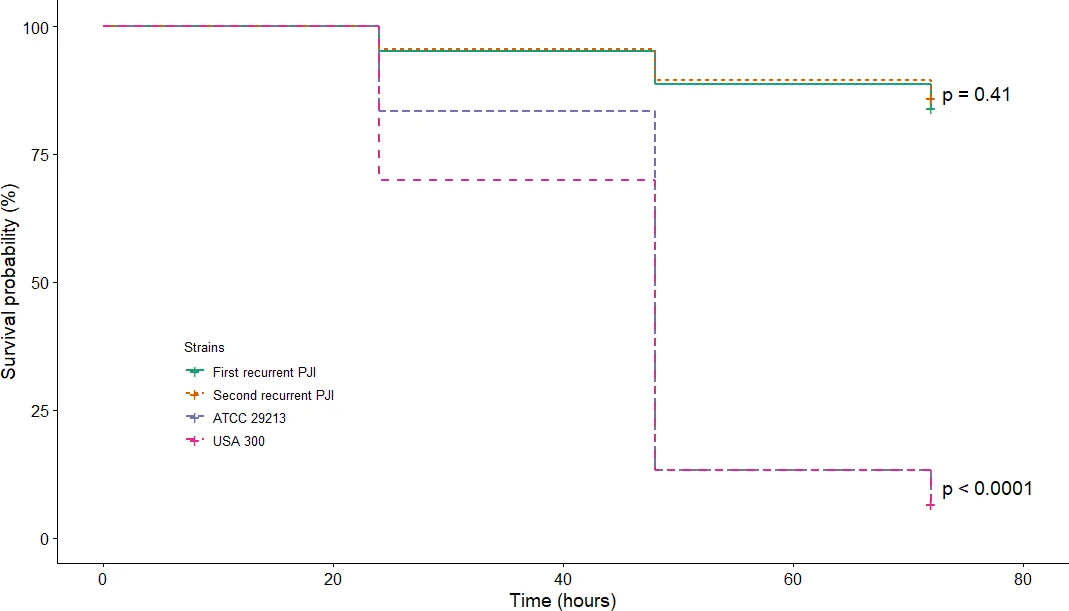
Kaplan-Meier survival curves *G. mellonella* larvae after infection with 2 × 106 CFU of strains isolated during the first recurrent PJI and during the second recurrent PJI.

To assess the fitness of strains recovered at different times of the TR-PJIs, growth rates were conducted. No difference was observed between strains isolated during the first and second TR-PJI episodes (Fig. S2). When analyzed collectively, second strains of TR-PJI exhibited a significantly lower biofilm-forming capacity than first strains (*P* < 0.001, Fig. 4). However, patient-level comparisons showed heterogeneous changes, with decreases in some patients (A and H), increases in others (D and G), and no significant differences in the remaining cases. Moreover, strains isolated during second TR-PJI tended to ferment glycerol, D-ribose, D-mannitol, D-trehalose, D-melezitose, amidon, D-turanose, and D-lyxose more frequently than strains isolated during first TR-PJI. On the contrary, fermented D-galactose and D-lactose (bovine origin) were fermented less frequently in the recurrent strains (not significant for all) (Fig. S3). Despite several genomic changes observed in the virulome analysis, we did not find a significant difference in mortality of larvae *Galleria mellonella* between strains isolated during the first and second TR-PJIs overall (*P* = 0.41) (Fig. 5) nor for strains that have acquired (*P* = 0.41 for patient D) or lost (*P* = 0.26 for patient C) virulence genes (data not shown). However, the virulence of all recurrent strains was reduced compared to control strains *S. aureus* ATCC 29213 and *S. aureus* USA 300 (*P* < 0.0001).

## DISCUSSION

The study of recurrent infections enables the understanding of intra-host evolution of chronic infections by longitudinally comparing strains isolated initially and during recurrences. Among our 13 patients with recurrent PJI, only eight (62%) had truly recurrent or persistent infections (TR-PJI), while five experienced reinfections with unrelated strains (FR-PJI). No single ST was dominant among our TR-PJI cases, indicating high genotypic diversity. Interestingly, we observed late infectious recurrences, occurring up to 35 months after the initial surgery.

Many studies have investigated the risk of recurrent infection with the same organism following medical and surgical management (19, 20, 31, 32). However, the definition of recurrence with the same organism was typically based only on the identification of the same bacterial species from two separate samples without genomic analysis to confirm the clonal lineage. In our study, one third of recurrent *S. aureus* infections were actually FR-PJI, not related to a failure of anti-infective treatment, but rather to recontamination during or after surgery. To the best of our knowledge, this is the second study to investigate recurrent *S. aureus* infections using genomic analysis to confirm whether they represented true recurrences or new infections. The first study reported four cases of recurrent *S. aureus* infection, of which three (75%) were attributed to TR-PJI, consistent with our results (13).

Two-stage exchange strategy involves the removal of prosthetic components and the debridement of the infected tissue, followed within 2 weeks to several months by reimplantation of a new prosthesis in a second stage (2). As all prosthetic material is completely removed without reimplantation during the first stage, the two-stage exchange is considered as the gold standard for minimizing the risk of persistent infection (2). However, this approach inherently requires multiple surgical procedures, each carrying a potential risk of new infection (33). Interestingly, a trend toward a higher frequency of two-stage procedures and prolonged (12-week) antibiotic therapy was observed in the FR-PJI group. It is worth noting that this study was conducted prior to the publication of the DATIPO randomized controlled trial, which demonstrated the superiority of a 12-week antibiotic regimen over a 6-week regimen in the management of PJIs (34). The trial showed a reduction in persistent infections within 2 years defined as the persistence or recurrence of infection with the initial causative organism, exhibiting a phenotypic antibiotic susceptibility profile identical to that observed at enrollment. However, some infections classified as recurrences in this trial with phenotypic approach were, in fact, new infections, which may have overestimated the risk of persistent infection in both arms.

Whole-genome sequencing (WGS) has become an essential tool for investigating clonal relationships between strains, particularly in the context of hospital outbreaks. However, defining clonality can be challenging, as bacterial genomes may undergo changes over time. Col et al. recently proposed a threshold of 25 whole-genome SNPs (wgSNPs) or 15 core-genome SNPs (cgSNPs) to distinguish genetically related strains from non-clonal ones (35). Among our eight patients with TR-PJIs, five had strains differing by >25 wgSNPs and two strains by >15 cgSNPs. However, the threshold proposed by Col et al. was based on a study investigating the risk of cross-transmission of MRSA between patients over a short period (<6 months). This cut-off does not appear applicable to our context since isolates were likely subjected to stronger selective pressures than in colonization settings and often evolved over longer time frames. Furthermore, sequencing and assembly methodologies may introduce biases, potentially generating artefactual SNPs that lead to an overestimation of genetic distances.

To date, only four studies have performed genomic comparison of *S. aureus* responsible for true recurrent osteoarticular infections (10–12, 36). Regarding genomic modifications, Loss et al. demonstrated the occurrence of a deletion of 9 base pairs in the *rpoB* gene coding for an RNA polymerase, the target of rifampicin, during a recurrent infection following arthroplasty (36). Mutations in this gene confer phenotypic resistance to rifampicin and hetero-resistance to vancomycin and daptomycin (37). Additionally, the H481Y mutation located in the *rpoB* gene has been associated with increased capsule synthesis and an enhanced stringent response. These features may be associated with an escape of the host immune response through reduced phagocytosis and persistence of the bacterium in a new niche (38). Suligoy et al. studied a strain of *S. aureus* responsible for chronic osteomyelitis and discovered an insertion in the *agrC* gene, leading to the loss of the type eight capsule, decreased expression of virulence factors, and increased biofilm production (12). While all of these studies reported phenotypic changes, such as altered ability of biofilm formation or virulence, only the studies by Suligoy et al. and Loss et al*.* identified genomic changes correlated with these phenotypic changes (12, 36). These revealed that some adaptive processes during chronic infections may occur at the transcriptional, translational, or post-translational level.

We conducted a comprehensive physiological analysis to identify potential patho-adaptations during chronic infection. Regarding antimicrobial resistance, we did not find any methicillin-resistant strains, and the percentages of strains resistant to rifampicin and fluoroquinolones, two antibiotics of choice in PJI, were 5.4 and 14.3%, respectively. Several factors could explain this low rate of resistance. First, the antibiotic resistance of *S. aureus* in France is relatively low. According to Santé Publique France in 2020, the percentages of hospital *S. aureus* resistant to methicillin, fluoroquinolones, and rifampicin were 14.0, 16.1, and 1.7%, respectively (39). However, considering the negative impact of methicillin resistance on the prognosis of *S. aureus* PJI (28), it was somewhat surprising that this resistance was not more prevalent in recurrent infections. The fitness cost associated with antibiotic resistance could explain this discrepancy (40). The occurrence of a point mutation in the target of an antibiotic or acquisition of resistance genes can have a global impact on bacterial transcription or translation, likely compromising the fitness and stress tolerance of resistant mutants (40, 41). A non-synonymous SNP (S486L) was found in the *rpoB* gene of a recurrent strain in a patient who had previously been treated with rifampicin in combination with trimethoprim/sulfamethoxazole. Rifampicin is a semisynthetic antibiotic that inhibits DNA-dependent RNA polymerase (42). It is a key treatment for staphylococcal PJI notably due to its anti-biofilm activity (2). Resistance to rifampicin arises by mutations in the *rpoB* gene, which encodes the β-subunit of the RNA polymerase enzyme (43). Given the presence of *rpoB* mutants within the bacterial inoculum at an estimated proportion of 1.10^6^ to 1.10^7^, the bactericidal activity of rifampicin against staphylococci is limited by the rapid emergence of resistance under selective pressure (44). Therefore, combination therapy is required to prevent the emergence of rifampicin resistance. In our case, rifampicin was initiated after a significant decrease in inoculum via surgery, administered at an adequate dose (>10 mg/kg/24 h), and combined with trimethoprim/sulfamethoxazole, as the subsequent emergence of rifampicin resistance remains unexplained. However, no antibiotic level monitoring was performed to verify adherence to treatment.

Biofilm is a structured microbial community adherent to a surface and embedded in a matrix composed of extracellular polysaccharides, proteins, and DNA (45). It is one of the most frequent mechanisms of persistence and treatment failure in *S. aureus* infections (46). Surprisingly, we observed a significant decrease in biofilm formation between the strains isolated during the first TR-PJI and those isolated during FR-PJI, contrasting with previous studies (10–12). In the study published by Muñoz-Gallego et al., six cases of persistent or relapsing PJI due to *S. aureus* were included, and no significant difference in biofilm formation assessed by crystal violet staining was observed between first and second strains (11). Suligoy et al. reported a case of chronic osteomyelitis of the tibia in a 20-year-old patient (12). They compared crystal violet biofilm formation of two strains of *S. aureus* isolated 13 months apart and showed that it was significantly greater for the strain isolated during recurrence compared to the initial strain. Trouillet-Assant et al. documented three cases of recurrent chronic infections (two PJIs and one tibial osteomyelitis involving osteosynthesis material) (10). The biofilm formation evaluated by crystal violet staining was significantly higher by the strains responsible for the two recurrent PJIs, whereas no significant difference was observed between the strains causing the osteomyelitis. Due to the small number of cases in each series, as well as in our own cohort, it is difficult to draw definitive conclusions on the evolution of the biofilm-forming capacity of *S. aureus* strains isolated in PJI over time. Furthermore, the storage conditions of our strains may have contributed to modifications in their biofilm-forming capacity. The isolates were maintained at room temperature for several years, and strains isolated during the first TR-PJI had a longer storage duration compared with those isolated during the second TR-PJI. Consequently, it is possible that the observed phenotypic differences reflect, at least in part, bacterial adaptation to prolonged *in vitro* storage conditions rather than true *in vivo* adaptation during persistence on the prosthetic surface. Further tests are needed using other dynamic methods to assess the ability to form biofilm.

Our study should be interpreted with caution due to its limitations. First, the limited cohort size (13 patients, including eight cases of recurrent PJI) constrains the robustness of the statistical analyses and the generalizability of the findings. Second, this is a retrospective study, and the strains isolated after subculturing depend on the quality of the surgical samples and the completeness of the initial storage. A founder effect cannot be ruled out during each sample, which potentially leads to a decrease in the microbial diversity observed and analyzed. Third, strains were initially stored at room temperature, which may have influenced the phenotypic changes observed, possibly reflecting bacterial evolution *in vitro*. Finally, we did not identify a clear correlation between the genomic alterations and the phenotypic changes observed. Moreover, we did not perform transcriptomic or proteomic analyses. Therefore, some of the phenotypic differences may be driven by transcriptional or translational regulatory mechanisms rather than by the genomic alterations identified.

Our study provides an overview of long-term patients with recurrent PJI due to *S. aureus* and the microevolution of strains isolated during chronic infections. Further studies are warranted to improve knowledge of the intra-host evolution of *S. aureus* during colonization/infection and the transition between acute and chronic infections, particularly in light of the alarming increase in antibiotic resistance and device-related infections.

## SUPPLEMENTAL MATERIAL

Supplemental tables and figure legends spectrum.04225-25-s0001.docx Tables S1 to S3 and Fig. S1 to S3.

Figure S1 spectrum.04225-25-s0002.tif Inhibition zone of erythromycin for strains isolated in patient B (a) and of rifampicin for strains isolated in patient C (b).

Figure S2 spectrum.04225-25-s0003.tif Representative growth curves of strains isolated during the first (green) and second (blue) recurrent PJIs.

Figure S3 spectrum.04225-25-s0004.tif Metabolism analysis using the API50CH gallery of strains isolated during the first and second recurrent PJIs. Mapping analysis was generated with R v 4.2.1 software.

ASM does not own the copyrights to Supplemental Material that may be linked to, or accessed through, an article. The authors have granted ASM a non-exclusive, world-wide license to publish the Supplemental Material files. Please contact the corresponding author directly for reuse.
