# Supplementary material for: In-host adaptation of Staphylococcus aureus during recurrent prosthetic joint infections: a retrospective longitudinal study
Source: Microbiol Spectr. 2026 Sep 16;14(10):e04225-25. doi: 10.1128/spectrum.04225-25 (PMC13689372; doi:10.1128/spectrum.04225-25)
Supplement: Supplementary file 1 [file spectrum.04225-25-s0001.docx]

Table S1. Allelic variation in the cgSNP profiles between strains from the first and second episodes of PJI in patients with TR-PJI.

|  | Median | Minimum | Maximum |
| --- | --- | --- | --- |
| Patient A | 16 | 16 | 17 |
| Patient B | 6 | 2 | 8 |
| Patient C | 9 | 8 | 12 |
| Patient D | 13 | 10 | 16 |
| Patient E | 8 | 7 | 9 |
| Patient F | 4 | 4 | 4 |
| Patient G | 10 | 8 | 11 |
| Patient H | 7 | 6 | 8 |

Table S2. Comparison of clinical characteristics and management between TR-PJI and FR-PJIR patients.

|  | TR-PJI n=8 (%) | FR-PJI n=5 (%) | *p ** |
| --- | --- | --- | --- |
| Mean (SD) age - years | 69,5 (13,6) | 68,8 (6,1) | 0.90 |
| Male sex | 4 (50) | 2 (40) | 1 |
| Charlson Comorbidity Index |  |  |  |
| 0 | 1 (12,5) | 0 | 0.71 |
| 1 to 2 | 1 (12,5) | 1 (20) |  |
| 3 to 4 | 3 (38) | 3 (60) |  |
| ≥ 5 | 3 (38) | 1 (20) |  |
| ASA score >2 | 4 (50) | 3 (60) | 1 |
| Obesity (BMI > 30 kg/m^2^) | 3 (38) | 3 (60) | 0.59 |
| Diabetes mellitus | 0 | 1 (20) | 0.38 |
| Congestive heart failure | 3 (38) | 2 (40) | 1 |
| Cerebrovascular disease* | 1 (12,5) | 0 | 1 |
| Liver cirrhosis | 2 (25) | 0 | 0.49 |
| Chronic renal disease | 1 (12,5) | 0 | 1 |
| Affected joint |  |  |  |
| Hip | 4 (50) | 2 (40) | 1 |
| Knee | 4 (50) | 3 (60) | 1 |
| Number of previous surgeries ^a^ |  |  |  |
| 0 | 5 (62,5) | 1 (20) | 0.14 |
| 1 | 0 | 1 (20) |  |
| ≥ 2 | 3 (38) | 3 (60) |  |
| Mean delay from primary implantation of prothesis to PJI (SD) - months ^a^ | 85,9 (66,7) | 133,8 (106,2) | 0.41 |
| Type of infection according to the time after last surgery | |  |  |
| Early postoperative (<4 weeks) | 2 (25) | 1 (20) |  |
| Delayed postoperative (>4 weeks) | 3 (38) | 3 (60) | 0.59 |
| Hematogenous origin | 3 (38) | 1 (20) |  |
| Surgery ^a^ |  |  |  |
| DAIR | 5 (62,5) | 1 (20) | 0.27 |
| 1-stage exchange | 1 (12,5) | 0 | 1 |
| 2-stage exchange | 1 (12,5) | 4 (80) | **0.03** |
| Antimicrobial therapy ^a^ |  |  |  |
| Rifampicin | 5 (62,5) | 4 (80) | 1 |
| Fluoroquinolones | 4 (50) | 3 (60) | 1 |
| Cefazoline | 1 (12,5) | 0 | 1 |
| Trimetoprim-sulfamethoxazole | 1 (12,5) | 2 (40) | 1 |
| Cloxacillin | 1 (12,5) | 0 | 1 |
| Fusidic acid | 0 | 1 (20) | 1 |
| Amoxicillin-Clavulanic acid∞ | 1 (12,5) | 0 | 1 |
| Duration of antimicrobial therapy ^a^ | |  |  |
| 6 weeks | 5 (62,5) | 0 | 0.08 |
| 12 weeks | 3 (38) | 5 (100) | 0.08 |

Abbreviations : ASA, American society of anesthesiologists; BMI, Body mass index ; DAIR, Debridement antibiotics and implant retention; FR-PJI, False recurrent prosthetic joint infection; PJI, prosthetic joint infection; SD, standard deviation; TR-PJI, True recurrent prosthetic joint infection

^a^ Of the first recurrent PJI

∞ Indefinite chronic antibiotic suppression regimen

* Fisher’s exact test for quantitative variable and Student’s *t* or Mann-Whitney U test for quantitative variable

Table S3. Distance matrix of SNP analysis between strains isolated from the same patient and a reference strain with the same ST.

Patient A

|  | A-I | A-II | A-III |
| --- | --- | --- | --- |
| A-I | ## | 67 | 67 |
| A-II | 67 | ## | 2 |
| A-III | 67 | 2 | ## |

Patient B

|  | B-I | B-II | B-III | B-IV | B-V | B-VI |
| --- | --- | --- | --- | --- | --- | --- |
| B-I | ## | 11 | 20 | 18 | 21 | 16 |
| B-II | 11 | ## | 17 | 15 | 18 | 13 |
| B-III | 20 | 17 | ## | 10 | 13 | 8 |
| B-IV | 18 | 15 | 10 | ## | 11 | 6 |
| B-V | 21 | 18 | 13 | 11 | ## | 5 |
| B-VI | 16 | 13 | 8 | 6 | 5 | ## |

Patient C

|  | C-I | C-II | C-III | C-IV |
| --- | --- | --- | --- | --- |
| C-I | ## | 13 | 21 | 34 |
| C-II | 13 | ## | 22 | 35 |
| C-III | 21 | 22 | ## | 19 |
| C-IV | 34 | 35 | 19 | ## |

Patient D

|  | D-I | D-II | D-III |
| --- | --- | --- | --- |
| D-I | ## | 23 | 28 |
| D-II | 23 | ## | 25 |
| D-III | 28 | 25 | ## |

Patient E

|  | E-I | E-II | E-III |
| --- | --- | --- | --- |
| E-I | ## | 18 | 35 |
| E-II | 18 | ## | 29 |
| E-III | 35 | 29 | ## |

Patient F

|  | F-I | F-II |
| --- | --- | --- |
| F-I | ## | 17 |
| F-II | 17 | ## |

Patient G

|  | G-I | G-II | G-III | G-IV |
| --- | --- | --- | --- | --- |
| G-I | ## | 23 | 18 | 18 |
| G-II | 23 | ## | 15 | 15 |
| G-III | 18 | 15 | ## | 2 |
| G-IV | 18 | 15 | 2 | ## |

Patient H

|  | H-I | H-II | H-III | H-IV |
| --- | --- | --- | --- | --- |
| H-I | ## | 3 | 13 | 11 |
| H-II | 3 | ## | 10 | 8 |
| H-III | 13 | 10 | ## | 2 |
| H-IV | 11 | 8 | 2 | ## |

**Supplemental Figure Captions**

Figure S1. Inhibition zone of Erythromycin for strains isolated in patient B (a) and of Rifampicin

for strains isolated in patient C (b).

Figure S2. Representative growth curves of strains isolated during the first recurrent PJI (green) and during the second recurrent PJI (blue).

Figure S3. Metabolism analysis using API50CH gallery of strains isolated during the first recurrent PJI and during the second recurrent PJI. Mapping analysis was generated with R v 4.2.1 software
